# Is Remodelling of Corticospinal Tract Terminations Originating in the Intact Hemisphere Associated with Recovery following Transient Ischaemic Stroke in the Rat?

**DOI:** 10.1371/journal.pone.0152176

**Published:** 2016-03-25

**Authors:** Emma J. Mitchell, Deborah Dewar, David J Maxwell

**Affiliations:** Institute of Neuroscience and Psychology, College of Medicine, Veterinary Medicine and Life Sciences, University of Glasgow, Glasgow, United Kingdom; INSERM U894, FRANCE

## Abstract

Following large strokes that encompass the cerebral cortex, it has been suggested that the corticospinal tract originating from the non-ischaemic hemisphere reorganises its pattern of terminal arborisation within the spinal cord to compensate for loss of function. However many strokes in humans predominantly affect subcortical structures with minimal involvement of the cerebral cortex. The aim of the present study was to determine whether remodelling of corticospinal terminals arising from the non-ischaemic hemisphere was associated with spontaneous recovery in rats with subcortical infarcts. Rats were subjected to transient middle cerebral artery occlusion or sham surgery and 28 days later, when animals exhibited functional recovery, cholera toxin b subunit was injected into the contralesional, intact forelimb motor cortex in order to anterogradely label terminals within cervical spinal cord segments. Infarcts were limited to subcortical structures and resulted in partial loss of corticospinal tract axons from the ischaemic hemisphere. Quantitative analysis revealed there was no significant difference in the numbers of terminals on the contralesional side of the spinal grey matter between ischaemic and sham rats. The results indicate that significant remodelling of the corticospinal tract from the non-ischaemic hemisphere is not associated with functional recovery in animals with subcortical infarcts.

## Introduction

Stroke is the leading cause of neurological disability in the adult population and while spontaneous recovery of neurological function can occur in some patients, improvement is often limited and many live with significant and permanent disability. Reorganisation of surviving neural networks has been implicated in recovery of sensorimotor function following stroke [1]. However, the precise mechanisms of injury-induced brain plasticity are poorly understood and greater insight into these processes is required for the development of novel therapeutic approaches. Abnormally enhanced activity in the contralesional cortex has been reported in patients who survive a stroke which indicates the potential involvement of contralesional networks in processes leading to functional recovery [2,3,4,5]. Rodent models of stroke and anatomical tracing methods have been used to assess reorganisation of networks originating in the contralesional cortex. Results indicate that sprouting of axons occurs in the spinal cord, with the suggestion that these replace synaptic terminals lost from the denervated side. For example, following permanent middle cerebral artery occlusion [6,7], destruction of the primary motor cortex [8] or unilateral pyramidotomy [9], corticospinal tract (CST) axons originating from the contralesional hemisphere have been reported to sprout into the denervated spinal grey matter. Furthermore, manipulating the extent of CST sprouting from the non-ischaemic hemisphere influences sensorimotor outcome. For example, blocking the neurite growth inhibitor Nogo-A after experimental stroke increases sprouting and improves performance in motor tasks [10] while knockdown of plasminogen reduces sprouting and impairs performance in motor tasks [11]. Studies of this type support the notion that the extent of sprouting in the spinal cord underpins the amount of functional recovery after stroke involving the CST.

Despite reports of CST fibre sprouting in rodent models of stroke, it still remains to be established whether new CST axonal terminals are also formed. Increased staining of the synaptic-specific protein, synaptophysin, in the spinal cord following experimental stroke in mice, is indicative of new terminals being formed in the denervated side [12]. Further evidence comes from injection of a retrograde transynaptic tracer into the peripheral muscles of the impaired forelimb of rats subjected to permanent middle cerebral artery occlusion which resulted in increased labelling of the contralesional cortex, when compared with sham animals. The enhanced neuronal connection between the intact contralesional cortex and peripheral tissues is consistent with the formation of new synapses, at least in a model of permanent focal cerebral ischaemia which typically involves both cortical and subcortical infarcts [7,13].

Ischaemic stroke is a highly heterogeneous condition in that the nature of the arterial occlusion and extent of reperfusion dictate both the size and location of the infarct and therefore the degree of functional impairment and recovery. In humans, strokes can affect either or both the cerebral cortex and subcortical regions and this heterogeneity can be examined by using appropriate animal models. It has been estimated that less than 15% of all strokes involve cortical infarcts while the majority of strokes are subcortical [14, 15, 16, 17]. Previous studies of structural reorganisation within the spinal cord in rodent models of stroke have principally used those models in which there are either large cortical [8, 10] or both cortical and subcortical [7, 13] infarcts. To our knowledge the issue of whether subcortical infarcts lead to reorganisation of axon terminations in the spinal cord from the non-infarcted hemisphere has not been explored.

**Fig 1:**
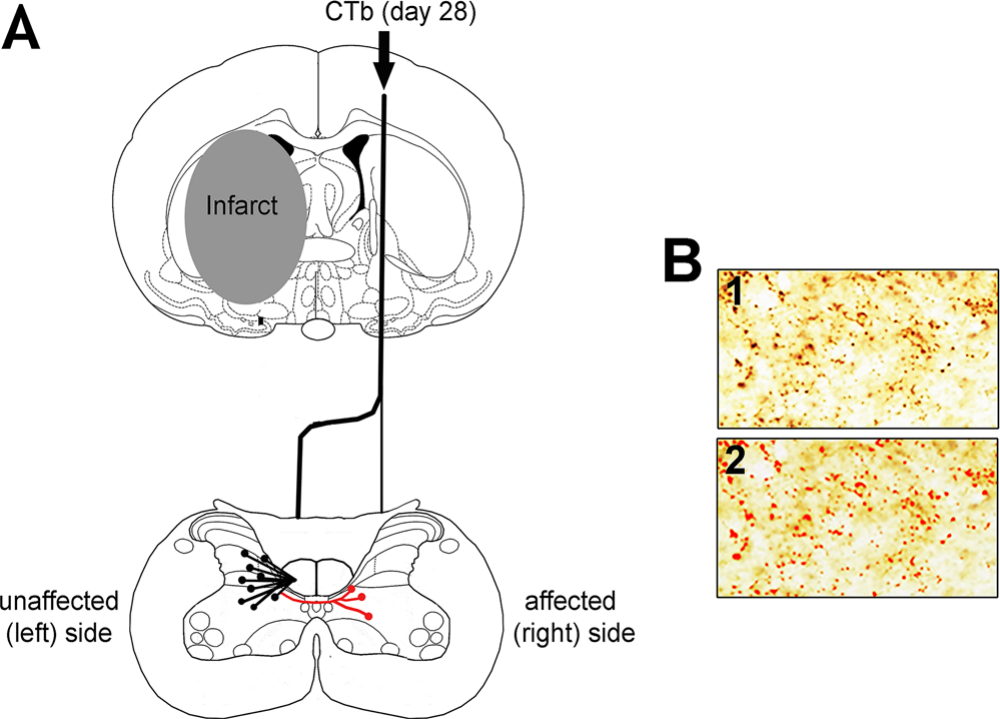
Experimental methods. A: Schematic diagram illustrating the experimental design. Twenty eight days following left MCAO, the CST arising from the non-ischaemic (right) hemisphere was anterogradely labelled with CTb. To assess for terminal remodelling, we quantified CTb-immunoreactive terminals in both sides of the cervical spinal cord. The black lines represent the predicted normal termination pattern of the labelled CST in an intact rat (terminals are present contralateral and also, but to a lesser extent, ipsilateral to the injection). Red lines represent potential new termination patterns that may arise after recovery from stroke. B: Automatic detection of CTb-immunoreactive terminals. Box 1 shows labelled terminals revealed with an immunoperoxidase reaction. Box 2 shows the same terminals detected using Image-Pro software (red).

The aim of the current study was therefore to determine whether the number of CST terminals in the denervated side of the spinal cord changed in association with functional recovery after subcortical stroke. We used a model of transient focal ischaemia in the rat which produced subcortical infarcts and cholera toxin b (CTb) subunit was injected into the intact, non-ischaemic forelimb motor cortex (Fig 1) at a time when rats exhibited spontaneous recovery of sensorimotor deficits to measure axonal terminations in the cervical region of the spinal cord.

## Materials and Methods

### Experimental design

All procedures were conducted under licence from the UK Home Office, in accordance with the Animals (Scientific Procedures) Act (1986) and approved by the University of Glasgow Animal Welfare and Ethical Review Panel. Anaesthesia was induced by inhalation of isoflurane for surgical procedures and euthanasia was performed by injection of sodium pentobarbitone and exsanguination.

**Fig 2:**
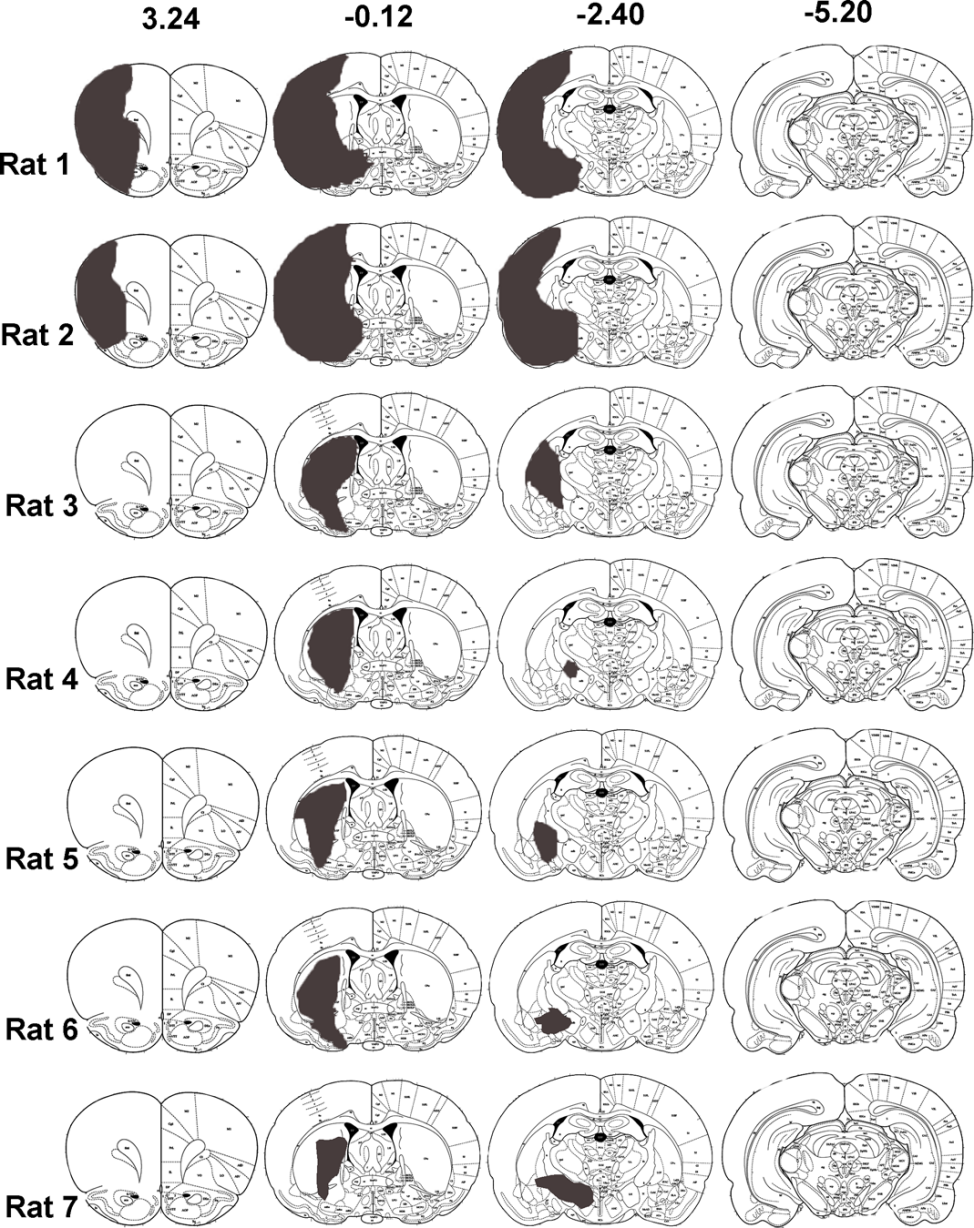
Distribution of infarcts induced by 60 min MCAO and reperfusion for 7 days. A separate group of rats to those used for CTb injections were subjected to 60 min MCAO and brains processed 7 days later for histological assessment. Paraffin sections at 8 pre-determined coronal planes through the extent of MCA territory were stained with haematoxylin and eosin and examined by light microscopy. Infarcts for each rat were transcribed onto atlas plate diagrams (grey areas). Bregma co-ordinates (from 24) are shown on the top row for 4 of the 8 coronal planes examined. Five out of 7 rats exhibited infarcts confined to subcortical regions with no evidence of damage in the cerebral cortex. Only 2 of the 7 rats exhibited infarcts that included the cerebral cortex.

Initially a pilot study was performed in which histological examination of 4% paraformaldehyde perfused- fixed brains from 7 rats was used to determine the anatomical distribution of infarcts induced by 60 min transient left middle cerebral artery occlusion (MCAO) (Fig 2). The main study included 4 rats subjected to MCAO that had subcortical but not cortical infarcts, as defined by T2 MRI scanning at 7 days, and 5 rats subjected to sham surgery. Neurological scoring and sensorimotor testing were conducted on all rats prior to surgery (day-1) and after surgery for 28 days for the assessment of functional deficit and recovery. At day 28, all rats received a stereotaxic injection of CTb into the forelimb area of the right sensorimotor cortex (Fig 1A). To determine the extent of axonal loss in the CST protein kinase C gamma (PKC-γ) labelling in the dorsal columns was performed. To assess terminal remodelling after MCAO, CTb-immunoreactive terminals in both sides of the cervical spinal cord were quantified. All image analysis was performed with the experimenter blinded to the identity of the rat.

### Transient middle cerebral artery occlusion

MCAO was performed for 60 min using a method of intraluminal vascular occlusion [18]. This duration of MCAO was based on results of the pilot study, whereby haematoxylin and eosin staining was used to characterise the extent and location of the infarct at 7 days following MCAO in 7 rats. Five out of the 7 rats were found to exhibit infarcts limited to subcortical structures, particularly the basal ganglia and internal capsule, and only 2 rats exhibited infarcts that extended into the lateral cortex (Fig 2). Thus, MCAO for 60 min was deemed a suitable technique for modelling subcortical ischaemic stroke. Briefly, rats were anaesthetised (2–3% isoflurane in oxygen) and artificially ventilated. Following exposure of the left common carotid artery bifurcation, a 20mm 3–0 filament with a blunted tip (~0.3mm diameter) was inserted into the external carotid artery and advanced along the lumen of the internal carotid artery until it blocked the origin of the middle cerebral artery. The filament was withdrawn after 60 minutes to allow for reperfusion. Sham operated animals were subjected to the same procedure with the exception of the advancement of the filament. Following withdrawal of anaesthesia animals regained consciousness and their health status monitored throughout the survival period.

### Assessment of sensorimotor function

Animals were assessed prior to MCAO and at 1, 2, 3, 7, 14, 21 and 28 days post-MCAO by using a 33-point neurological score modified from a previous report [19]. This comprised a battery of 11 tests that measured a range of motor behaviours including grip strength, forepaw reaching, forepaw reflexes, paw placement, motility and circling. The adhesive label test, which measures the latency to remove an adhesive tab from the ventral surface of each forepaw, was performed prior to and at 3, 7, 14, 21 and 28 days after MCAO [20]. Each session for this test consisted of 4 trials and the order of tab application (left, right) was randomised. For each trial, the difference in removal time between the affected (right) and unaffected (left) paw was calculated to prevent the overall activity of the rats from affecting performance [21]. For both tests, performances were averaged for all of the animals in each group and data expressed as the mean ± standard deviation.

### Quantification of infarct volume

Seven days following MCAO, rats were anaesthetised (as above) and transferred to a Bruker Biospec 7T/30cm (Ettlingen, Germany) MRI scanner with a gradient coil (121mmID, 400mT/m) and a 72 mm birdcage resonator. A RARE T_2_-weighted sequence was then acquired (TE = 72.7ms, TR = 5086.1ms, matrix = 256x256, 16 coronal slices; 0.75mm thick). Infarcted areas were manually delineated on each of the 16 slices using ImageJ software and the infarct volume (mm^3^) was calculated by multiplying the total area across the 16 slices by slice thickness (0.75mm). Infarct volume is presented as the mean ± standard deviation.

### Anterograde labelling of the CST arising from the non-ischaemic hemisphere

At 28 days following MCAO, a solution of CTb (Sigma-Aldrich, Co., Poole, UK) was injected into the forelimb motor cortex of the right, non-ischaemic hemisphere (stereotaxic location derived from [22]). Following induction of anaesthesia (described above), the rat was placed in a stereotaxic frame, the skull was exposed and 2 burr holes were made at the following coordinates: (I) anterioposterior +1mm, mediolateral -3mm, dorsoventral -1.5mm; (II) anterioposterior -0.5mm, mediolateral -1.5mm, dorsoventral -1.5mm, relative to Bregma. A glass micropipette with a tip diameter of 20μm filled with 1% CTb in sterile distilled water was inserted into the brain and CTb was injected at 4 standardised points (200nl per point) between the 2 burr holes using a Pico injector (10 ms pulses at 20 psi, World Precision Instruments, Sarasota, FL, USA). For each injection the micropipette remained in place for 5 minutes to prevent backflow of tracer. The scalp was then sutured and the animals were recovered.

### Tissue preparation

Four days following CTb injection (32 days after MCAO), rats were anaesthetised (pentobarbitone, 1ml of 200mg/ml; intraperitoneal) and transcardially perfused with saline followed by 4% paraformaldehyde in 0.1M phosphate buffer. The spinal cord and brain were removed and post-fixed for 8h at 4°C. The brain was kept in a fixative containing 30% sucrose for 24 hours and cut coronally (100μm) using a freezing microtome (Leica, UK). Segments C3-C8 from the cervical spinal cord were cut into transverse sections (60μm) with a vibratome (Oxford instruments, Technical products international Inc. USA).

### Assessment of CST integrity

The intensity of PKC-γγ immunoreactivity in the dorsal columns was measured in order to examine whether MCAO led to loss of CST axons projecting from the ischaemic hemisphere. PKC-γ is a signalling kinase present in axons of the dorsal CST [23] and has been employed to determine the extent of CST axonal loss in stroke models [10]. For each animal, PKC-γ immunostaining in 2 transverse sections (60 μm) from segment C8 of the cervical spinal cord was assessed. Firstly, sections were treated with 50% ethanol for 30 minutes to enhance antibody penetration. They were then incubated with anti- PKC-γ for 72 hours (Table 1) followed by donkey anti-rabbit Alexa 488 secondary antibody (24 hours). Sections were rinsed in 0.1M phosphate-buffered saline and mounted with anti-fade mounting medium (Vectasheild Vector Laboratories, Peterborough, UK) on glass slides. The dorsal columns were imaged using a BioRad Radiance 2100 confocal laser scanning microscope (BioRad, Hemmel Hempstead, UK) at 20x magnification (zoom factor of 0.9 at 0.5 μm intervals). The intensity of PKC-γ labelling was measured using Image J software and expressed as a ratio of the affected (right) versus unaffected (left) side to control for inter-animal variation. PKC-γ labelling intensity ratios were averaged for all of the rats in each group.

**Table 1:** Summary of primary and secondary antibody combinations.

	Primary antibody combination	Concentration	Supplier	Secondary antibody combination	Sequential reaction
1	rb. PKC-γ	1:500	List Biological Laboratories, Campell, CA	Alexa488	
2	gt. CTb	1:5000	List Biological Laboratories, Campell, CA	Biotinylated IgG	Avidin HRP (1:1000) + DAB
3	gp. VGLUT1	1:5000	Millipore, Harlow, UK	Dylight649	
	gt. CTb	1:5000	List Biological Laboratories, Campell, CA	Alexa488	

1: Assessment of PKC- γ labelling in the dorsal columns of segment C8. 2: Visualisation of cortical CTb injection sites and labelled terminals in segments C3, C5 and C7. 3: Assessment of VGLUT1 immunoreactivity in labelled terminals in (segment C4). Abbreviations: gt = goat; gp = guinea pig; mo = mouse; rb = rabbit; Rh.Red = rhodamine red

### Identification of cortical CTb injection sites

CTb injection sites in the non-ischaemic cerebral cortex were revealed with an immunoperoxidase reaction (Table 1). Brain sections were incubated in goat anti-CTb for 48 hours followed by biotinylated anti-goat immunoglobulin gamma (IgG) for 3 hours. Sections were then treated with avidin horseradish peroxidise for 1 hour and reacted (~ 15 minutes) with hydrogen peroxide and 3, 3’-diaminobenzidine (DAB) to reveal immunoreactivity. Injection sites were viewed with transmission light microscopy and photographed digitally (AxioVIsion 4.8 software, Zeiss, Germany) and the extent of each injection site was mapped onto standard coronal section diagrams obtained from the stereotaxic rat brain atlas [24].

### Assessment of VGLUT1 immunoreactivity in CTb-labelled axonal swellings

CST axonal terminals are enriched with vesicular glutamate transporter 1 [25], therefore the presence of VGLUT1 was used to verify that CTb-labelled axonal swellings were terminals. Three rats from each of the sham and MCAO groups were randomly selected by an individual who was blind to the rat identity and sections from segment C4 were reacted with antibodies against CTb and VGLUT1 (Table 1). Using confocal microscopy, fields containing CTb-labelled terminals were scanned with a 40x oil-immersion lens (zoom factor of 2 at 0.5μm intervals). For each section (3–4 per animal), 4 fields (3 from the unaffected (left) side and 1 from the affected (right) side of the spinal grey matter) with 100 x100μm scanning area were obtained. Using Neurolucida software (MBF Bioscience, Colchester, VT, USA), stacks were initially viewed in a grid (10 x 10 μm^2^) so that only CTb-immunoreactivity was visible. For each square, a CTb-labelled terminal closest to the bottom right corner was marked, and then the marked terminals were examined in the blue channel to assess for co-labelling of VGLUT1. The percentage of double-labelled CTb terminals as a proportion of the total CTb-labelled terminal count was calculated for each animal. This value was averaged for the three animals in each group.

### Quantification of CTb-immunoreactive terminals

Sections from segments C3, C5 and C7 in all rats were processed for DAB immunoreactivity in the same manner as those sections used to identify CTb injection sites (Table 1). For each animal, DAB-labelled terminals were counted on 3 sections each from segments C3, C5 and C7. For each section, a tiled (x40 magnified) image was acquired and a region of interest (0.9mm x 1mm) was applied to each side of the grey matter. Each region of interest was aligned with the ventromedian fissure (the midline) and encompassed the deep dorsal horn and intermediate grey matter. This area of the grey matter is where the majority of premotor interneurons are located [26] and any recovery as a consequence of sprouting is likely to involve the formation of *de novo* CST contacts with premotor neurons. We did not include the ventral horn in our analysis because CST terminals are sparse in the rat ventral horn and do not directly influence motor neurons [27, 28]. Furthermore, previous studies have reported CST collaterals to sprout into the denervated dorsal and intermediate grey matter after permanent MCAO and unilateral pyramidotomy [6, 7, 9]. Three different methods were used to assess CTb terminal labelling: 1) the number of labelled terminals within each box was determined automatically using Image-Pro Premier software (MediaCybernetics: Fig 1B); 2) the total CTb-positive surface area (μm^2^) within each box was measured; 3) terminals adjacent to the central canal of C5 sections (box size 200 μm x 300 μm for each side) were counted manually using Image J (National Institutes of Health, USA). Terminal counts and CTb-positive surface areas in sham and MCAO rats were compared for each side of the cervical spinal cord separately. To minimise inter-animal variation that may have occurred due to subtle differences in the CTb injection volume or location, terminal counts and CTb-positive surface areas were expressed as ratios: affected/unaffected (ipsilateral to injection/contralateral) and the ratios in sham and MCAO rats were compared.

### Statistical analyses

Neurological scores and adhesive label test data were analysed separately within sham and MCAO groups to compare matched pre-surgery baseline and post-surgery measurements. This was done in order to detect functional impairment and recovery within the MCAO group. Data were analysed using Friedman’s test for matched repeated measures and Dunn’s post hoc test was applied to compare performances at day 0 (pre-surgery) versus day 3 and day 7 to assess the degree of functional impairment and day 0 versus day 28 to assess the extent of recovery. Mann-Whitney test was used to analyse in differences in PKC-γ immunostaining and CTb labelling between sham and MCAO groups. A value of P<0.05 was taken to indicate a statistically significant difference.

## Results

### Sensorimotor dysfunction and spontaneous recovery following MCAO

**Fig 3:**
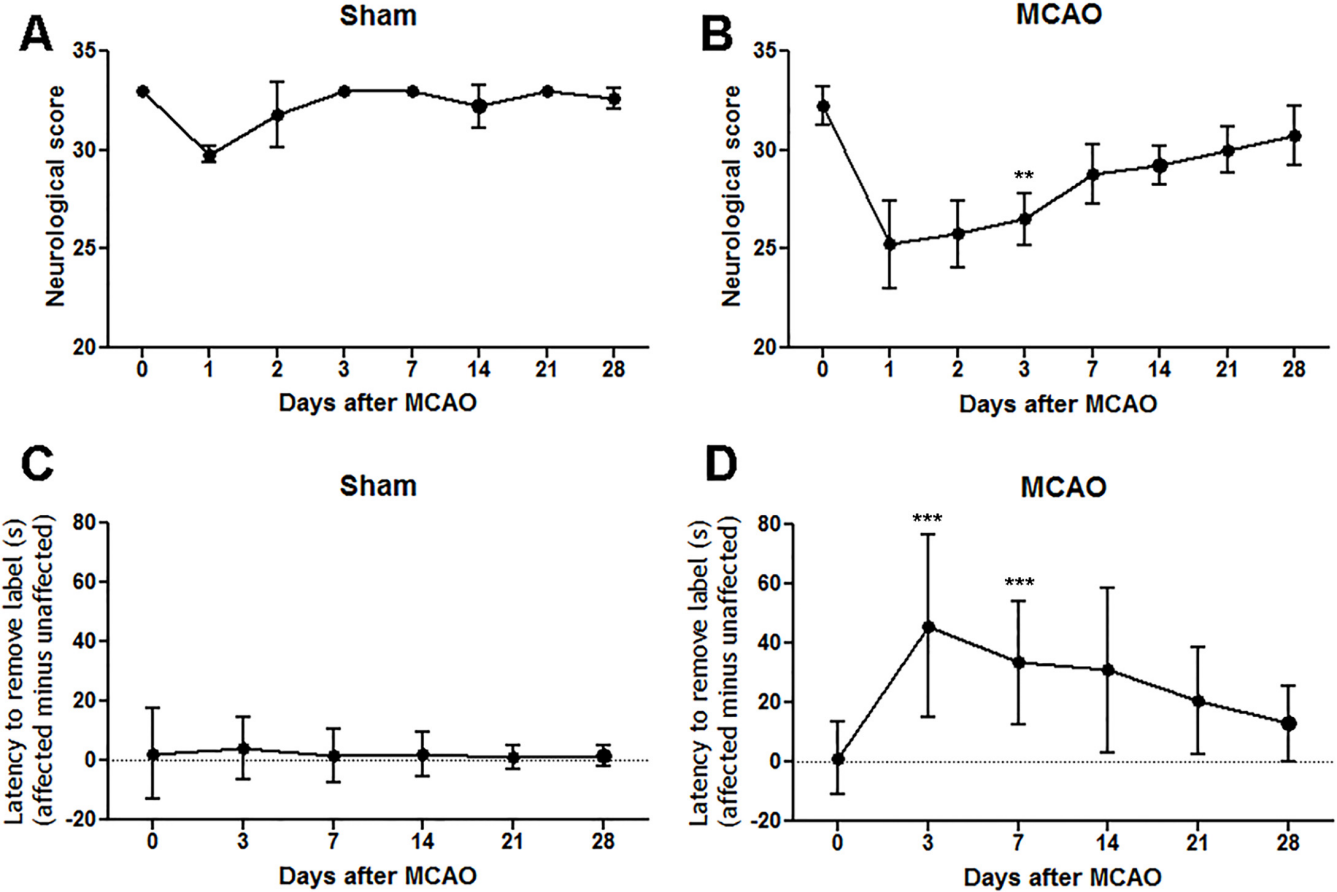
Functional deficits and recovery following MCAO. A: Sham rats exhibited neurological scores that were not significantly altered compared to their pre-surgery baseline score (day 0). B: Following MCAO, rats exhibited a significant reduction in neurological score at 3 days followed by gradual recovery such that at 28 days there was no difference compared to their pre-MCAO baseline scores. C: Sham rats did not exhibit alterations in the removal difference times between the affected versus unaffected forepaws at any timepoint after surgery compared to pre-surgery baseline. D: MCAO resulted in significantly increased removal difference times between the affected and unaffected forepaws at 3 and 7 days and this deficit gradually recovered such that at 28 days there was no difference compared to pre-MCAO baseline measurements. Data analysed by Friedman’s test followed by Dunn’s multiple comparisons test; **p<0.001 versus day 0; ***p<0.0001 versus day 0.

Compared to pre-surgery baseline, sham-operated rats had lower neurological scores in the first 1–2 days following the procedure, most likely due to the acute effects of anaesthesia and surgical procedures, but from 3 days onwards the scores were no different from baseline (Fig 3A). The first two days were therefore excluded from the analysis of the neurological data from MCAO rats since scores are affected by the surgical procedure and anaesthesia. At 3 days after MCAO, rats had significantly lower neurological scores compared to pre-surgery baseline indicating that infarcts in these animals were associated with functional deficits. There was gradual improvement over time such that at 28 days neurological scores of MCAO rats were not different from their pre-MCAO baseline scores, which is indicative of recovery (Fig 3B). For the adhesive label test of forelimb sensorimotor function in sham-operated rats, the difference in adhesive removal time between the affected and unaffected forelimbs was not altered at any post-surgery time point compared to pre-surgery baseline (Fig 3C). Rats with MCAO exhibited significant impairment on this test at 3 and 7 days after surgery compared to pre-surgery baseline. Thereafter this bilateral asymmetry gradually became less pronounced until there was no significant difference between the removal time measured at 28 days after MCAO and pre-surgery baseline indicating that there was recovery of forelimb sensorimotor function (Fig 3D).

### Infarcts and loss of CST fibres following MCAO

**Fig 4:**
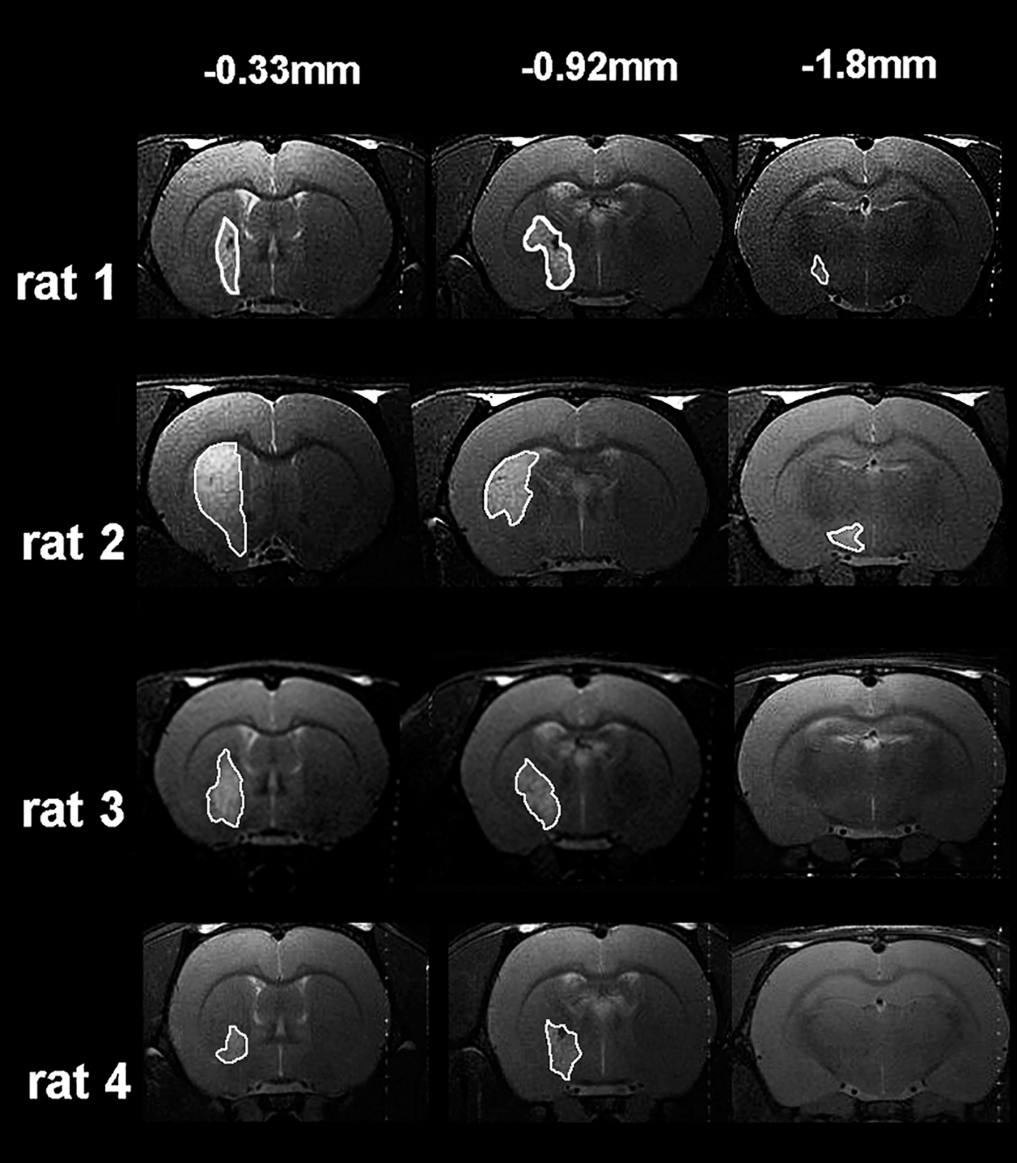
Subcortical infarcts at 7 days following MCAO. T_2_-weighted MRI images from all rats subjected to MCAO. The distance from Bregma (mm), [24] is shown on the top line. Note that in all rats, infarcted tissue (the hyperintense regions outlined in white) is confined to subcortical structures, particularly within the striatum and does not extend to cortical areas.

**Fig 5:**
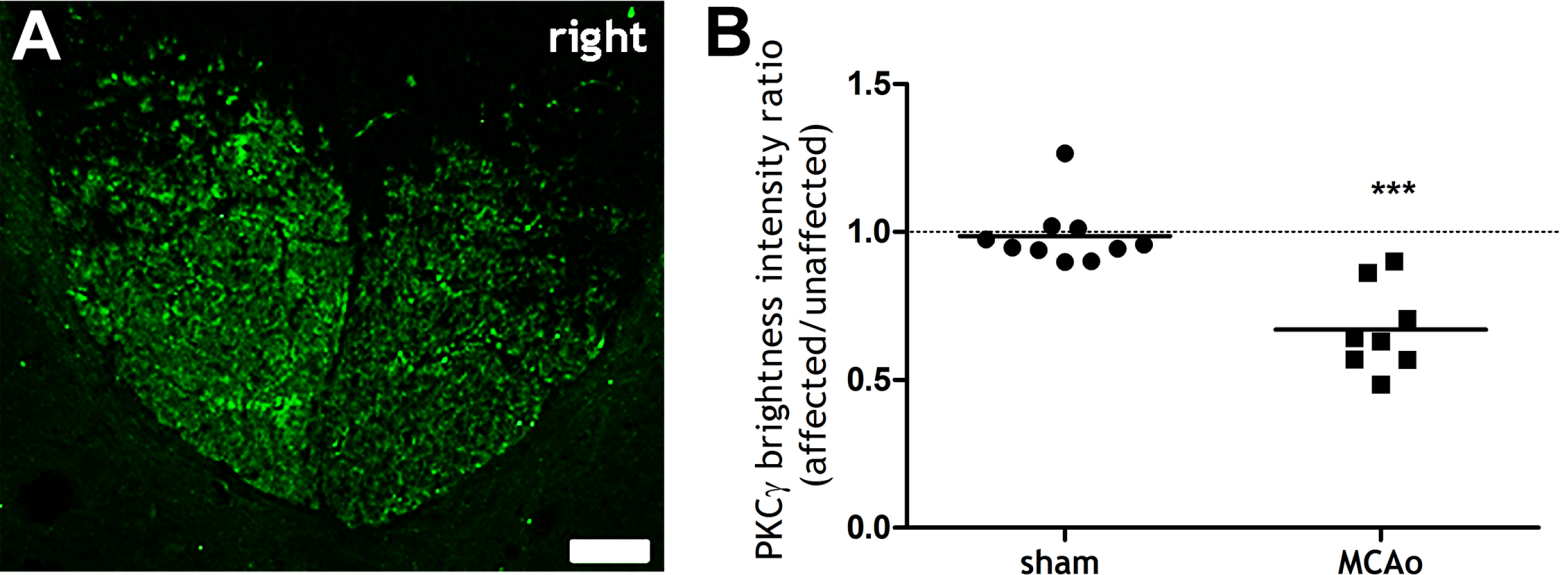
Axonal loss in the dorsal columns at 28 days following MCAO. A: Projected confocal microscope image of the dorsal columns (C8) from a rat with MCAO immunolabelled for PKC-γ. Note the reduced PKC-γ immunoreactivity in the affected (right) side, indicative of CST axonal loss (Scale bar = 50μm). B: PKC-γ brightness intensity, expressed as a ratio of affected (right) side/unaffected (left) side, was significantly reduced in the MCAO group compared to the sham group. Each data point represents a spinal section (2 per rat). The dashed horizontal line indicates 1, which denotes symmetrical PKC-γ immunoreactivity between affected and unaffected sides. ***p<0.0001, Mann-Whitney test MCAO compared to sham.

All rats subjected to functional assessments exhibited subcortical infarcts, mainly affecting the striatal region, with no detectable infarction in cortical areas (Fig 4A). Visual inspection of all 64 MRI slices obtained from the 4 rats subjected to MCAO revealed subcortical infarcts to be present in 20 slices and cortical infarcts to be present in 0 slices. The lesion volume was 45.0 ± 18.0 mm^3^ (mean ± SD; range 33.0 to 70.3 mm^3^). The extent of injury to the CST was examined at the level of the cervical spinal cord using immunoreactivity for PKC-γ. In MCAO rats, partial loss of PKC-γ contralateral to the ischaemic hemisphere (affected/right side) was observed (Fig 5A). The mean PKC-γ brightness intensity ratio (affected versus unaffected side) was significantly reduced in the MCAO group compared with the sham group (Fig 5B).

### CTb injection sites

**Fig 6:**
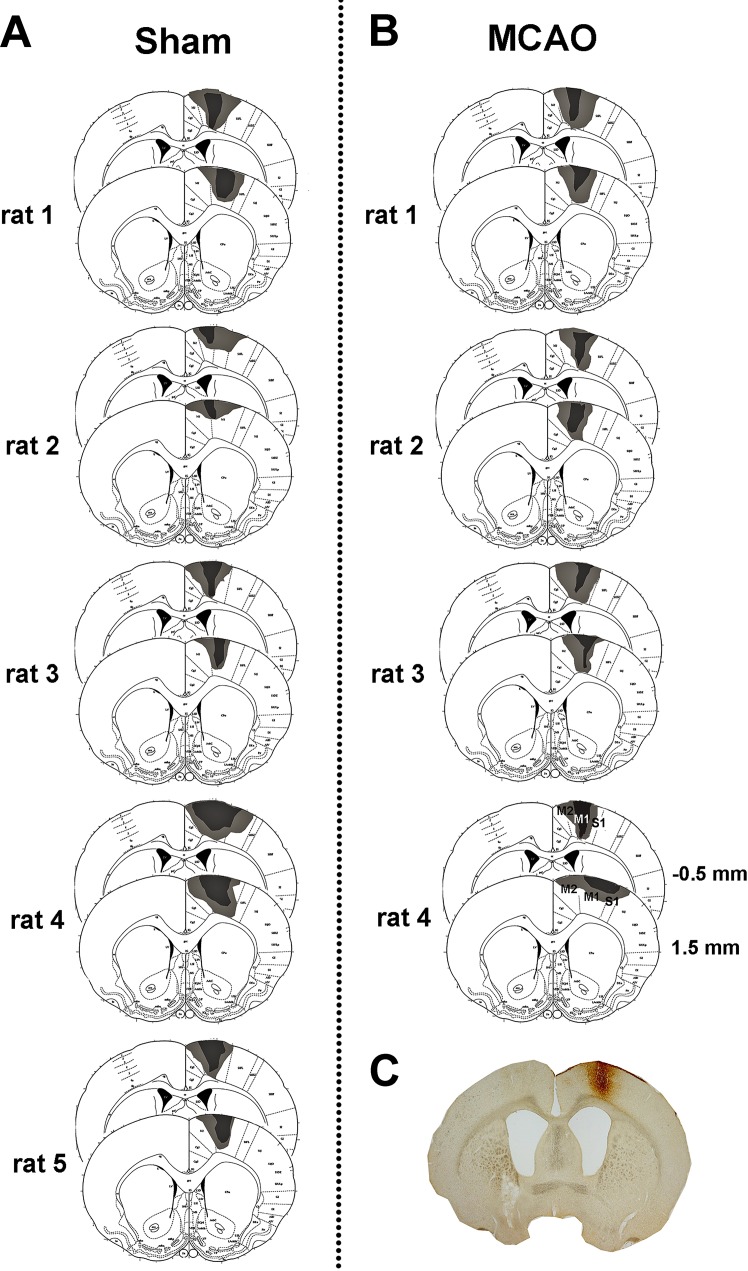
CTb injection sites in the contralesional cortex. A: Reconstruction of CTb- injection sites relative to the location of the forelimb motor cortex for sham rats (based on atlas of Paxinos and Watson [24] and stereotaxic coordinates defined by Neafsey et al., [22]) Numerical values indicate the distance from Bregma (mm). B: A similar sequence of images for MCAO rats. The dark shading shows the core of the injection and the light shading shows the spread surrounding the injection. Injection sites included primary (M1) and secondary (M2) motor cortices as well as the primary sensory cortex (S1). C: A photomicrograph of a coronal section illustrating a CTb injection site in a MCAO rat (rat 4) (0.5mm relative to Bregma).

In sham and MCAO rats, CTb injection sites were confined to the contralesional cortex (left side), as revealed by immunoperoxidase reactions (Fig 6). The core of each injection was focussed within the primary and secondary motor cortex and primary sensory cortex (Fig 6).

### CTb-immunoreactive variscosities in the spinal cord contained VGLUT1

**Fig 7:**
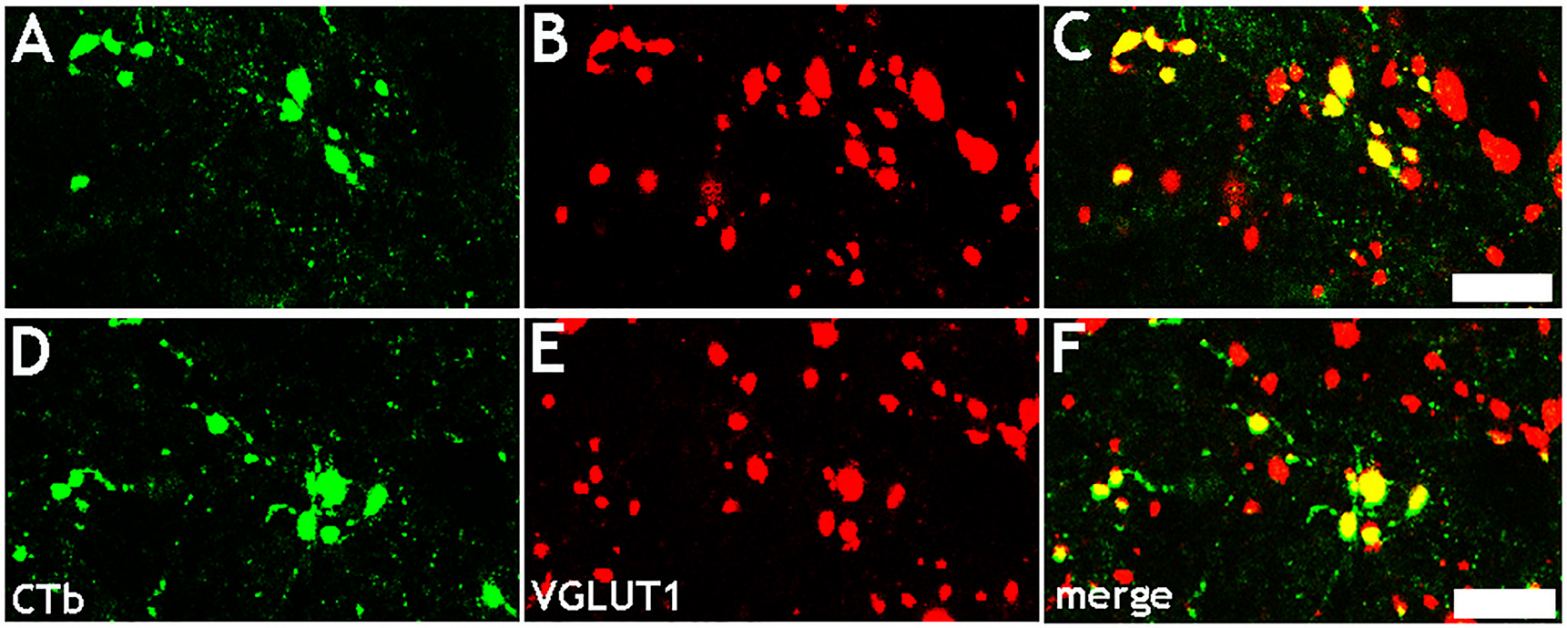
Co-localisation of VGLUT1 with CTb-labelled axonal swellings. Confocal microscope images showing co-expression of VGLUT1 with CTb-labelled axonal swellings in the spinal cord originating from the non-ischaemic hemisphere. A: Single optical section showing CTb-labelled axonal swellings (green) in a sham rat. B: immunoreactivity in the same plane for VGLUT1 (red). C: Merged image. D-F: A similar series is also shown for a MCAO rat. Scale bars = 5μm.

In both sham and MCAO rats, CTb-labelled axonal swellings originating from the sensorimotor cortex of the contralesional hemisphere contained VGLUT1 (Fig 7). In the sham group there were 385 ± 97 (mean ± SD) CTb-labelled terminals per animal and 93 ± 4% of these contained VGLUT1. In the MCAO group there were 720 ± 161 (mean ± SD) CTb-labelled terminals per animal and 96 ± 1% of these contained VGLUT1.

### MCAO did not alter the laminar distribution pattern of CTb-immunoreactive terminals

**Fig 8:**
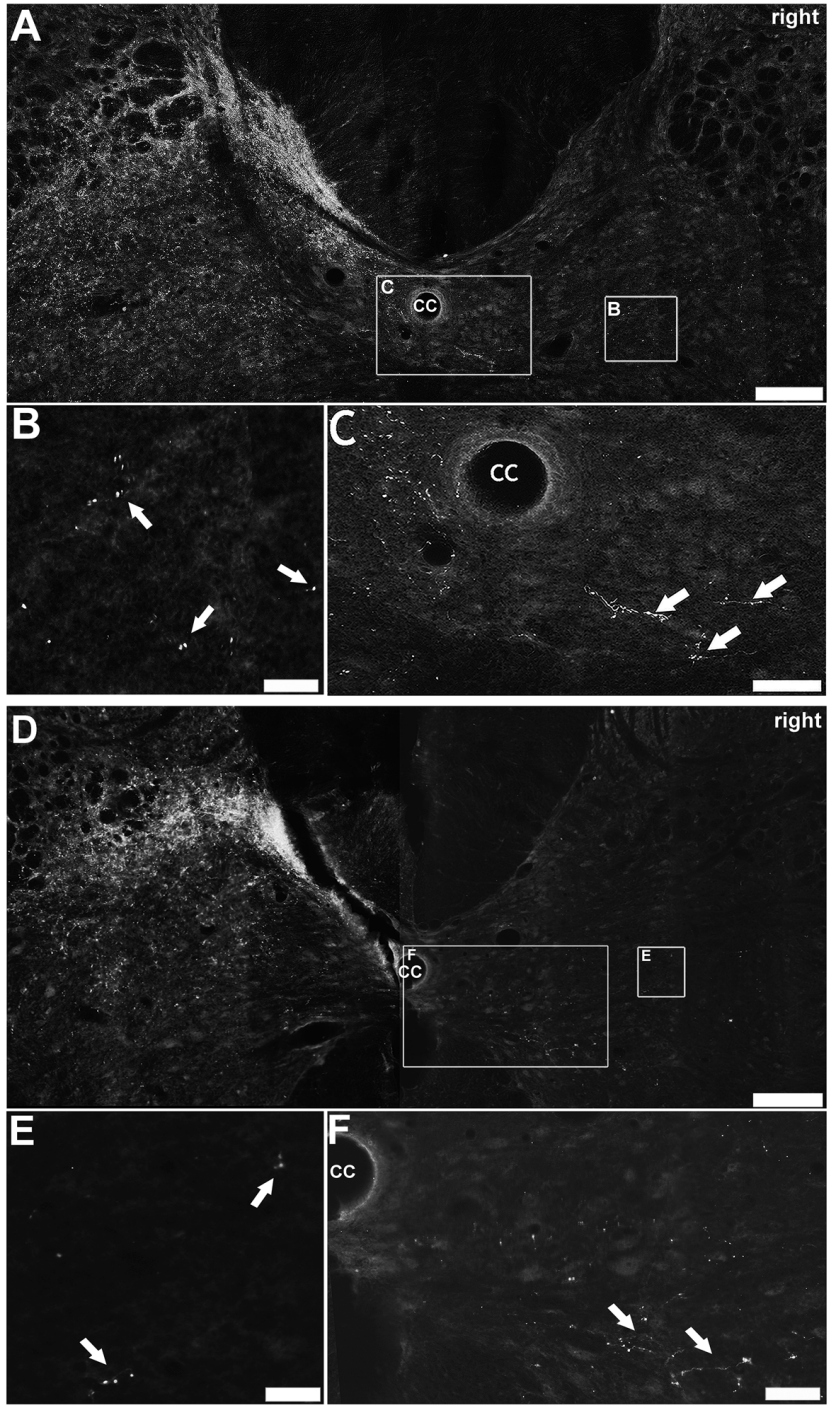
Distribution of CTb-labelled terminals. A: CTb-immunoreactive terminals in a C3 section from a sham rat. The majority of terminals were located in the side contralateral to the injection site (unaffected/left side) at the medial base of the dorsal horn. Terminals were also dispersed throughout the intermediate grey matter of the right side (B). Occasionally, fibres and terminals were detected adjacent to the central canal (C). B & C are magnified images of the insets in A. A similar series of images are shown for a MCAO rat (D, E, F). Scale bar in A & D = 150μm; Scale bar in B, C, E & F = 50μm.

Labelled terminals in the cervical spinal cord were similarly distributed in all sham (Fig 8A) and MCAO (Fig 8D) rats. In both groups terminals were primarily located in the grey matter contralateral to the injection site (unaffected/left side). They were most numerous in the medial region of the deep dorsal horn (laminae III-VI). Fewer terminals were present within the intermediate grey matter and only occasional terminals were found in the ventral horn. Occasionally terminals were observed ipsilateral to the injection site (affected/ right side), mainly in the intermediate grey matter (See Fig 8B & Fig 8E, for sham and MCAO examples, respectively) consistent with the termination pattern of uncrossed axons described previously. Within the affected/ right side of the grey matter, a very small number of terminals and fibres were detected immediately adjacent to the central canal, in both sham (Fig 8C) and MCAO (Fig 8F) rats. Because of the proximity to the midline, they may be ramifications of midline crossing fibres. This occurrence was very rare in both sham and MCAO rats and was only detected in 5 out of 15 and 3 out of 12 sections, respectively.

### MCAO did not significantly alter the numbers of CTb-labelled terminals

**Fig 9:**
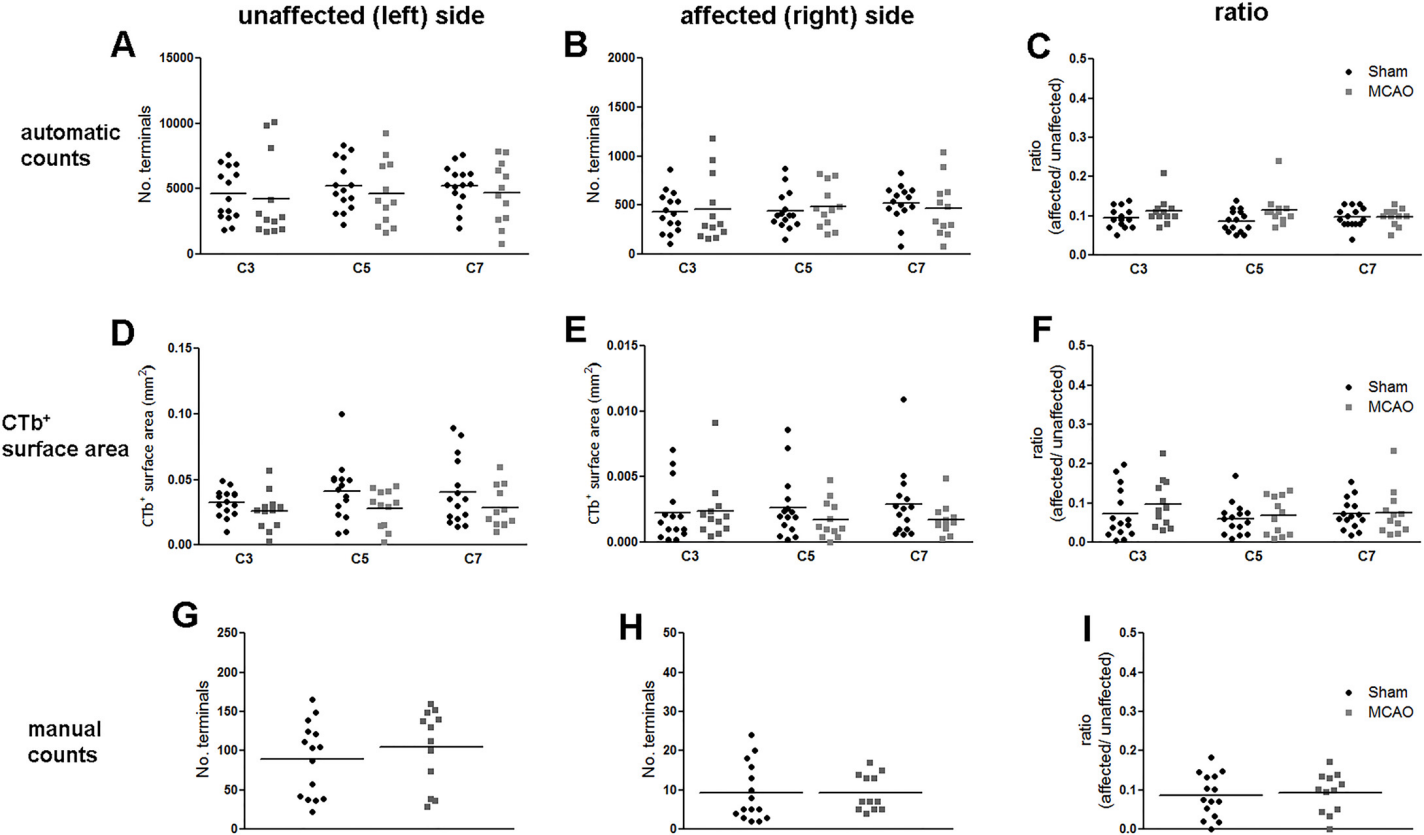
Quantification of CTb-immunoreactivity the cervical spinal cord. For each of the three quantification methods, results are presented for the unaffected (left) side, affected (right) side and the ratio (affected/unaffected). A,B,C: Automatically counted terminals. D,E,F: CTb^+^ surface area. G,H,I: manually counted terminals. There were no significant differences between sham and MCAO rats (p>0.05). Data represent mean ± SD. Each point represents counts from a single spinal section (3 per rat).

For each segment analysed by automatic counting software (C3, C5, C7), the mean number of terminals in the unaffected (left) side was not significantly different between sham and MCAO rats (Fig 9A). Similarly, for all analysed segments, the mean number of terminals in the affected (right) side was not significantly different between sham and MCAO rats (Fig 9B). Terminal counts expressed as a ratio: affected/unaffected (ipsilateral to injection/contralateral) were not significantly different between sham and MCAO rats (Fig 9C). The mean CTb-positive surface area (mm^2^) within the unaffected (left) side of the grey matter for all segments analysed (C3, C5, C7), was not significantly different between sham and MCAO rats (Fig 9D). Similarly, there were no significant differences between sham and MCAO rats for the CTb-positive surface area in the affected (right) side (Fig 9E) or for the CTb-positive surface area ratios (Fig 9F). Terminals immediately adjacent to the central canal in the C5 segment were counted manually. However, there were no significant differences between sham and MCAO rats in the number of terminals in the affected (right) side (Fig 9G), the number of terminals in the unaffected (left) side (Fig 9H) or the affected:unaffected ratio (Fig 9I).

## Discussion

The rats subjected to MCAO included in this this study exhibited subcortical (but not cortical) infarcts and this was associated with loss of approximately 35% of CST axons originating from the ischaemic hemisphere. Compared to pre-surgery baseline, rats exhibited sensorimotor deficits and impaired neurological function in the early phase after MCAO. However, animals with MCAO recovered over time such that sensorimotor function had recovered to baseline levels at the time when spinal cord tissue was examined. Despite the functional recovery demonstrated by MCAO rats, the number of axon terminals in the cervical spinal cord which originated in the contralesional cortex was not altered compared to shams. In all rats, CTb-immunoreactive terminals were located predominantly within the grey matter contralateral to the injection site (unaffected/left side), particularly within the medial base of the dorsal horn (lamina III to VI), as described previously [25, 29]. A much smaller number of diffusely scattered labelled terminals was also present ipsilateral to the injection site (affected/right side), throughout the intermediate grey matter. Although we cannot rule out the possibility that terminals observed in the ipsilateral (affected) side of the cord in both sham and MCAO rats originated from the crossed component of the CST, these terminals are most likely to be components of the uncrossed CST which has been described previously [30]. Uncrossed CST pathways have been demonstrated to be capable of influencing motor neuron activity [31]. It is probable that most of the axonal swellings examined in this study are sites of synaptic interaction as they were universally immunoreactive for VGLUT 1 which is present in some types of glutamatergic presynaptic boutons, including those of the CST [25]. No significant differences were found between the number of labelled terminals within the affected or the unaffected sides of the cervical spinal cord in MCAO rats and the left and right sides of sham rats. Therefore the findings do not support the hypothesis that significant changes occur in the densities of synaptic terminals in the denervated side of cervical spinal segments at a time when rats with subcortical infarcts have achieved functional recovery. This was an exploratory study with small group sizes and one limitation is the the possibility of a Type II error. As far as we are aware there have not been previous studies of CTb-immunoreactive terminal counts in the spinal cord after subcortical infarcts in rats. Therefore there was no existing data which could be used prior to the study to calculate appropriate group sizes. Using the standard deviation of the automatic counts of CTb-immunoreactive terminals in the affected (right) side (segment C5) we have performed sample size calculations using power = 0.8 and α = 0.05 (StatMate, GraphPad Software, USA) for a potential future study of this type and design. It is difficult to predict what a biologically- or functionally-significant, as opposed to a statistically-significant change, in terminal number would be, but the n value that would be required in a future study to detect a 30% difference in hypothetical mean values between groups is n = 35, a 20% difference is n = 80 or a 10% difference n = 370. We cannot exclude the possibility that there may have been subtle changes in terminal reorganisation in response to subcortical infarcts and that these were of insufficient magnitude to be detected by the methodology we employed. For example, formation of *de novo* CST synaptic connections with commissural interneurons in the intact side of the cord could provide an alternative route for information to be conveyed from the contralesional hemisphere to the denervated half of the spinal cord, in a similar way to the synaptic remodelling that occurs following spinal damage to the CST [32]. It must also be acknowledged that the motor dysfunction observed following MCAO may not have been solely attributed to loss of CST axons from the ischaemic hemisphere. For instance, the internal capsule has extensive subcortical-cortical connections within the affected hemisphere and between the two hemispheres and lesioning of the internal capsule results in depressed activity of these remote interconnected structures [33]. Thus, it cannot be ruled out that diaschisis contributed to the motor deficits and that the resolution of diaschisis participated in the functional recovery.

An increased number of terminals in the affected half of the spinal cord in the context of an infarct involving the CST might have been predicted based on previous reports of axonal sprouting from the contralesional hemisphere to the denervated half of the spinal cord after MCAO [6,7] or destruction of the primary motor cortex [8] or unilateral pyramidotomy [9]. Previous studies have reported recovery of staining for the presynaptically-located protein, synaptophysin, in axons on the denervated side of the spinal cord [12] and such changes were associated with functional recovery in rats with cerebral lesions. In the current study the numbers of CTb-immunoreactive terminals in the cervical spinal cord in functionally-recovered rats with subcortical infarcts were not significantly different to that of sham-operated animals. One possible explanation for the seeming contradiction between our study and others is that the extent of terminal reorganisation of the contralesional CST depends on the size and location of the infarct. It is therefore interesting to compare our study with others which have used MCAO to induce ischaemic damage. Liu and colleagues reported sprouting of the intact contralesional CST after MCAO-induced infarcts [6] which, given the method and duration of arterial occlusion, typically encompass large regions of both the cerebral cortex and subcortical regions. A subsequent study from the same laboratory reported a similar pattern of sprouting in animals which had partial recovery of skilled forelimb reaching [12]. In both of these previous studies, animals were examined 28 days after MCAO and this is a similar time-point after MCAO to that examined in the current study. Therefore, it seems unlikely that we did not detect changes because the timeframe of our study was markedly different from these others. However, the location and severity of the infarcts in the current study were different from those studies. The infarcts produced in the current study were relatively small and located exclusively to subcortical regions. This contrasts with the size and typical distribution of infarcts in the aforementioned studies. Our study also contrasts with other reports of sprouting after the entire motor cortex was infarcted by means of photothrombosis [8] or the entire CST was physically ablated [9]. In our study approximately 75% of the CST, as measured by PKC−γ immunostaining of at the level of the pyramids, was preserved. Taken together the evidence thus raises the possibility that in situations where there is sparing of a relatively large proportion of the CST (as in the current study), fibres from the ischaemic hemisphere may predominantly mediate recovery. Contrastingly, after severe strokes that involve a large proportion, or all of the CST, ipsilesional plasticity may be limited and contralesional networks are recruited [34]. If this hypothesis is correct, then in strokes where only a minority of CST axons from the ischaemic hemisphere are destroyed, the remaining ipsilesional CST fibres may have a major role in mediating recovery. Interestingly, deactivation of the intact hemisphere with lidocaine following recovery from MCAO was found to reinstate the original deficits only in rats with large infarcts [35]. In human stroke survivors, the degree of acute ischaemic damage to the CST at the level of the internal capsule predicts the severity of the motor deficit present 3 months after the stroke [36], suggesting that the integrity of the CST from the ischaemic hemisphere may be closely linked to functional improvement. Recovery from stroke, whether involving large or small infarcts, is unlikely to be solely attributed to CST terminal reorganisation, and other systems are highly likely to be involved. For example, sprouting of the corticorubral tract in association with recovery has been reported [37], as has strengthening of reticulospinal tract input to motor neurons following transection of the CST [38, 39]. In addition to reorganisation at the level of the spinal cord, plastic changes throughout the damaged and/or intact hemisphere are likely to be involved [1].

In conclusion, we did not detect changes in the density of spinal synaptic terminals within ipsilesional cervical spinal segments of functionally recovered rats with subcortical infarcts. While interventions that enhance structural reorganisation in the spinal cord have been proposed as potential therapeutic approaches to enhance recovery after stroke, the heterogeneous nature of human stroke, in terms of infarct size and location, means it will be important to determine which patient groups such approaches might benefit.
