# Correction: Is Remodelling of Corticospinal Tract Terminations Originating in the Intact Hemisphere Associated with Recovery following Transient Ischaemic Stroke in the Rat?

**DOI:** 10.1371/journal.pone.0155665

**Published:** 2016-05-11

**Authors:** Emma J. Mitchell, Deborah Dewar, David J Maxwell

The following information is missing from the Funding section: This work was funded by the Medical Research Council (grant number MR/J50032X/1).
